# Phylogenetic reconstruction of dengue virus type 2 in Colombia

**DOI:** 10.1186/1743-422X-9-64

**Published:** 2012-03-09

**Authors:** Jairo A Méndez, José A Usme-Ciro, Cristina Domingo, Gloria J Rey, Juan A Sánchez, Antonio Tenorio, Juan C Gallego-Gomez

**Affiliations:** 1 Laboratorio de Virología, Instituto Nacional de Salud, Avenida/Calle 26 No. 51-20, Bogotá, D.C., Colombia; 2 Viral Vector Core and Gene Therapy, Neurosciences Group, Sede de Investigación Universitaria, Universidad de Antioquia, A.A. 1226 Medellín, Colombia; 3 Departamento de Ciencias Biológicas-Facultad de Ciencias, Laboratorio BIOMMAR, Universidad de los Andes, Carrera 1 No. 18a-10 Bloque J-309, Bogotá, D.C., Colombia; 4 Laboratorio de Arbovirus y Enfermedades Víricas Importadas, Centro Nacional de Microbiología, Instituto de Salud Carlos III, Carretera Majadahonda-Pozuelo Km2, Majadahonda, 28220 Madrid, Spain; 5 Robert Koch Institute, Nordufer 20, Berlin 13353, Germany

**Keywords:** Dengue virus, Phylogenetic, Serotype, Genotype, Evolution, Colombia

## Abstract

**Background:**

Dengue fever is perhaps the most important viral re-emergent disease especially in tropical and sub-tropical countries, affecting about 50 million people around the world yearly. In Colombia, dengue virus was first detected in 1971 and still remains as a major public health issue. Although four viral serotypes have been recurrently identified, dengue virus type 2 (DENV-2) has been involved in the most important outbreaks during the last 20 years, including 2010 when the fatality rate highly increased. As there are no major studies reviewing virus origin and genotype distribution in this country, the present study attempts to reconstruct the phylogenetic history of DENV-2 using a sequence analysis from a 224 bp PCR-amplified product corresponding to the carboxyl terminus of the envelope (E) gene from 48 Colombian isolates.

**Results:**

As expected, the oldest isolates belonged to the American genotype (subtype V), but the strains collected since 1990 represent the American/Asian genotype (subtype IIIb) as previously reported in different American countries. Interestingly, the introduction of this genotype coincides with the first report of dengue hemorrhagic fever in Colombia at the end of 1989 and the increase of cases during the next years.

**Conclusion:**

After replacement of the American genotype, several lineages of American/Asian subtype have rapidly spread all over the country evolving in new clades. Nevertheless, the direct association of these new variants in the raise of lethality rate observed during the last outbreak has to be demonstrated.

## Background

During the last few decades, the whole world has faced the re-emerging of different infectious diseases, being dengue one of the most important in terms of morbidity and mortality [1-5]. Dengue virus (DENV) is an arbovirus belonging to family *flaviviridae *and is responsible of a wide range of clinical manifestations in humans, including an acute self-limited flu-like illness known as dengue fever (DF) or a severe illness known as dengue hemorrhagic fever (DHF) characterized by a marked plasma leakage, which may progress to hypovolemic shock (dengue shock syndrome, DSS) with circulatory failure [1,3,4,6-8]. Nevertheless, changes observed in clinical manifestations (in terms of severity) during the few last years have obliged to redefine this classification according to the presence of alarm signs [4].

As usual in flavivirus, DENV is an enveloped virus with a positive sense ssRNA of about 11 kb coding a single open reading frame for three structural proteins, core (C), membrane (M) and envelope (E), and seven non-structural proteins (NS1, NS2A, NS2B, NS3, NS4A, NS4B, NS5). Based on serological analysis, DENV can be differentiated as four distinct serotypes (DENV-1, DENV-2, DENV-3 and DENV-4), which are capable of causing the disease. Although the exact mechanisms for DHF remains unclear, it is generally accepted that a secondary infection with a heterologous serotype increases the risk of severe manifestations as a consequence of the antibody dependent enhancement (ADE) mechanism proposed by Halsted in the 80's [7]. However, an alternative explanation to patho-physiology of DHF is the emergence and spread of virulent strains originated as part of an evolutionary process [2,9,10]. In fact, molecular epidemiology using nucleotide sequence analysis from the DENV genome has demonstrated the occurrence of genotype clades within each serotype [11-29], which highlights the important role of DENV itself in disease severity rather than immune enhancement [2,9,10].

The four serotypes of DENV have been circulating in the Americas since the early 1900's, generating only slight cases of DF and sporadic cases of severe disease [1,5,6,30]. It was not until 1981 when the first large epidemic of DHF occurred in Cuba and rapidly spread to Jamaica (1981-1982), Brazil (1986), and Venezuela (1989-1990) [1,2,5,8,9,12,23]. In Colombia, the first case of DHF was officially notified in December of 1989 from the village of Puerto Berrio (Antioquia department) [25,31]. Since then, DHF became endemic and lethal cases rapidly increased during the next years. Although co-circulation of serotypes was common in different countries, samples from these major outbreaks confirmed DENV-2 as the main responsible of DHF cases. In 1997, Rico-Hesse et al., demonstrated that DENV-2 isolated from DHF outbreaks in Jamaica and the Caribbean islands (and possibly Cuba) in 1981-1982 belonged to a new clade formerly named "Asian genotype", probably introduced from South Asia, where severe infection has been persistent since the middle of the past century [2,23]. To date, DENV-2 falls into seven subtypes (or genotypes) designed as Subtype I (Asian II), Subtype II, Subtype IIIa (Asian I), Subtype IIIb (American/Asian), Subtype IV (Cosmopolitan), Subtype V (American) and Sylvatic genotype [2,12,13]. Additionally, the existence of clades with distinctive geographical and temporal relationships has been suggested [23].

Historically, Colombia has been one of the most affected countries in the Americas with dengue epidemics [6,30,31]. In fact, during the last year it went through the largest dengue epidemic occurred in decades, with 157,152 cases notified (mostly DENV-2) and 217 deaths confirmed [32]. Nevertheless, there are no major studies regarding DENV-2 phylogenetic origin or genotype circulation and distribution [25]. Consequently, the present study tries to reconstruct phylogenetics of DENV-2 virus that has been circulating in Colombia during the 1980's in comparisons to the strains isolated since the emergence of DHF. In addition, this work describes the evolution of new clades during the last decade based on a partial nucleotide sequence of the envelope (E) gene.

## Results

### Virus recovery and confirmation

Forty-eight viruses obtained from symptomatic patients were isolated in mosquito cell culture and subsequently identified as DENV-2 serotype by monoclonal antibodies and confirmed by RT-PCR methods [33]. Isolates are listed in Table 1 indicating locality, isolation year, genotype and accession number.

**Table 1:** Colombian DENV-2 isolates sequenced and analyzed in the present study

ISOLATE	LOCALITY	ISOLATION YEAR	SUBTYPE/GENOTYPE	ACCESSION NUMBER
DENV-2/CO/186_Tolima/1982	Tolima	1982	V/American	JF906211

DENV-2/CO/299_Tolima/1983	Tolima	1983	V/American	JF906212

DENV-2/CO/298_Nariño/1983	Nariño	1983	V/American	JF906213

DENV-2/CO/183_Nariño/1985	Nariño	1985	V/American	JF906214

DENV-2/CO/184_Nariño/1985	Nariño	1985	V/American	JF906215

DENV-2/CO/348563_Nariño/1985	Nariño	1985	V/American	JF906216

DENV-2/CO/348561_Huila/1985	Huila	1985	V/American	JF906217

DENV-2/CO/350447_Tolima/1987	Tolima	1987	V/American	JF906218

DENV-2/CO/350446_Tolima/1987	Tolima	1987	V/American	JF906219

DENV-2/CO/351863_Cundinamarca/1988	Cundinamarca	1988	V/American	JF906220

DENV-2/CO/362091_Caqueta/1988	Caqueta	1988	V/American	JF906221

DENV-2/CO/350449_Tolima/1988	Tolima	1988	V/American	JF906222

DENV-2/CO/351861_Bolivar/1988	Bolivar	1988	IIIb/American- Asian	JF906223

DENV-2/CO/360281_Tolima/1992	Tolima	1992	IIIb/American-Asian	JF906224

DENV-2/CO/274_Santander/1997	Santander	1997	IIIb/American-Asian	JF906225

DENV-2/CO/271_Santander_1997	Santander	1997	IIIb/American-Asian	JF906226

DENV-2/CO/193_Tolima/1997	Tolima	1997	IIIb/American-Asian	JF906227

DENV-2/CO/201_Arauca/1998	Arauca	1998	IIIb/American-Asian	JF906228

DENV-2/CO/202_Tolima/1998	Tolima	1998	IIIb/American-Asian	JF906229

DENV-2/CO/272_Cundinamarca/1998	Cundinamarca	1998	IIIb/American-Asian	JF906230

DENV-2/CO/377717_Cundinamarca/1999	Cundinamarca	1999	IIIb/American-Asian	JF906231

DENV-2/CO/218/2001	NA	2001	IIIb/American-Asian	JF906232

DENV-2/CO/219/2001	NA	2001	IIIb/American-Asian	JF906233

DENV-2/CO/355_Guaviare/2002	Guaviare	2002	V/American	JF906234

DENV-2/CO/222/2002	NA	2002	IIIb/American-Asian	JF906235

DENV-2/CO/357_Tolima/2002	Tolima	2002	IIIb/American-Asian	JF906236

DENV-2/CO/376_SanAndres/2003	San Andres	2003	IIIb/American-Asian	JF906237

DENV-2/CO/371_Cauca/2003	Cauca	2003	IIIb/American-Asian	JF906238

DENV-2/CO/392_Guajira/2003	Guajira	2003	IIIb/American-Asian	JF906239

DENV-2/CO/383_Guajira/2003	Guajira	2003	IIIb/American-Asian	JF906240

DENV-2/CO/384_Tolima/2003	Tolima	2003	IIIb/American-Asian	JF906241

DENV-2/CO/378_Tolima/2003	Tolima	2003	IIIb/American-Asian	JF906242

DENV-2/CO/397/2004	NA	2004	IIIb/American-Asian	JF906243

DENV-2/CO/402_Putumayo/2004	Putumayo	2004	IIIb/American-Asian	JF906244

DENV-2/CO/399_Guajira/2004	Guajira	2004	IIIb/American-Asian	JF906245

DENV-2/CO/241_Guaviare/2005	Guaviare	2005	IIIb/American-Asian	JF906246

DENV-2/CO/413037_Amazonas/2010	Amazonas	2010	IIIb/American-Asian	JF906247

DENV-2/CO/412968_Santander/2010	Santander	2010	IIIb/American-Asian	JF906248

DENV-2/CO/413036_Amazonas/2010	Amazonas	2010	IIIb/American-Asian	JF906249

DENV-2/CO/412597_Amazonas/2010	Amazonas	2010	IIIb/American-Asian	JF906250

DENV-2/CO/413034_Amazonas/2010	Amazonas	2010	IIIb/American-Asian	JF906251

DENV-2/CO/413033_Amazonas/2010	Amazonas	2010	IIIb/American-Asian	JF906252

DENV-2/CO/408339_Valle/2010	Valle	2010	IIIb/American-Asian	JF906253

DENV-2/CO/410149_Cesar/2010	Cesar	2010	IIIb/American-Asian	JF906254

DENV-2/CO/408243_Risaralda/2010	Risaralda	2010	IIIb/American-Asian	JF906255

DENV-2/CO/408338_Valle/2010	Valle	2010	IIIb/American-Asian	JF906256

DENV-2/CO/V-163/1961	NA	1961	I/Asian II	JF906257

DENV-2/CO/256/1971	NA	1971	I/Asian II	JF906258

NA = Not Available

### Phylogenetic reconstruction of DENV-2

Sequences from the carboxyl terminus of the envelope (E) gene from the 48 Colombian DENV-2 isolates were aligned in CLUSTAL W [34,35] and compared with 28 previously reported sequences elsewhere, resulting in a trivial alignment as long as there were not insertions or deletions (INDELS). Although the use of the whole E protein gene is highly recommended to reconstruct DENV phylogenies, the 224 bp sequence used in our study has been demonstrated to be useful to infer phylogenetic relationships while the topology is fully maintained [26,27,29].

**Figure 1:**
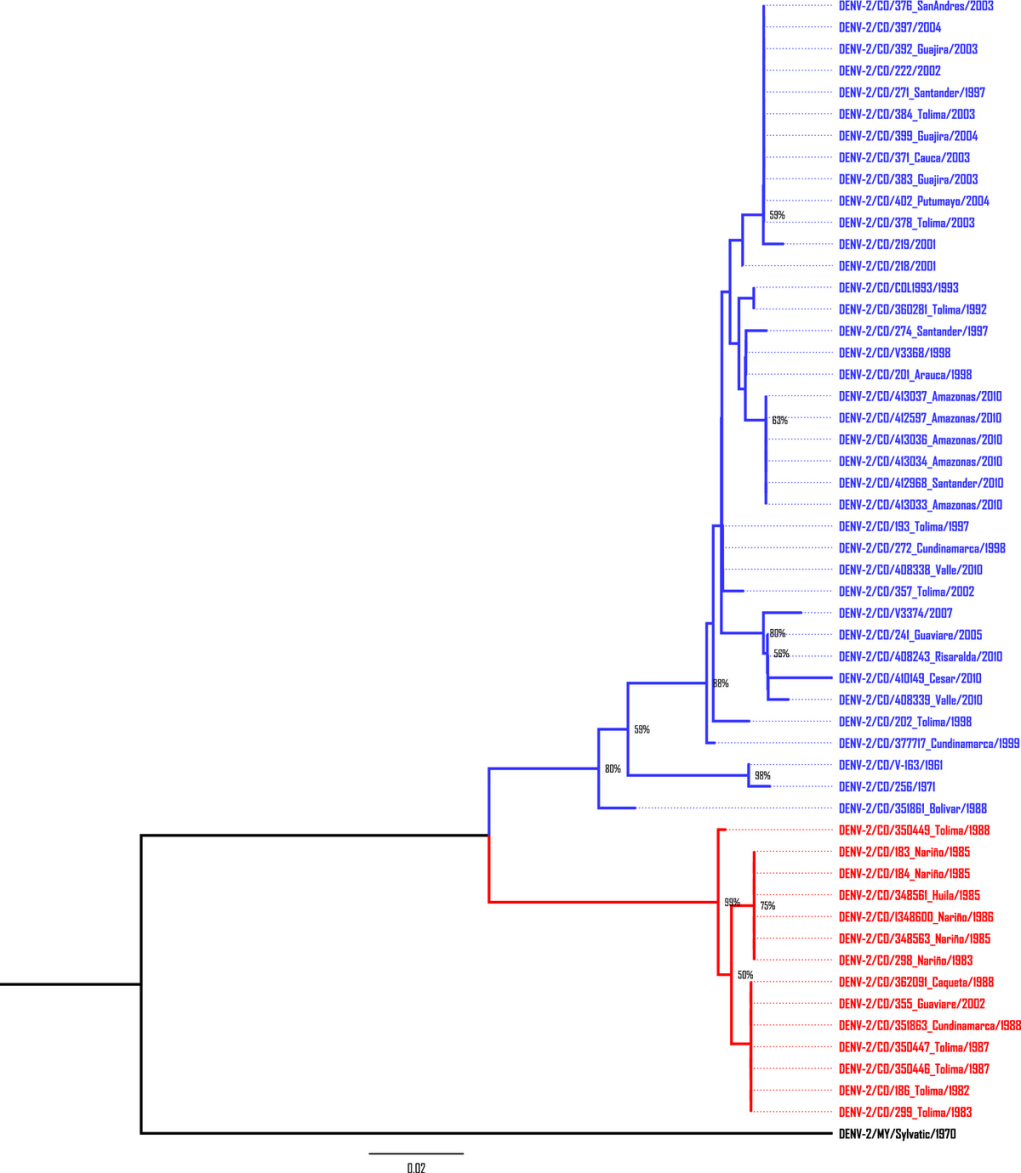
**Evolutionary relationships of Colombian DENV-2**. Phylogenetic tree of 52 DENV-2 Colombian isolates was computed using the Maximum Likelihood method. Nodes with significant Neighbor- Joining bootstrap values (≥ 50) are indicated. The tree is drawn to scale, with branch lengths in the same units as those of the evolutionary distances used to infer the phylogenetic tree. Two well defined clades are shown in red and blue. The tree is rooted using one Sylvatic sequence.

The Maximum Likelihood (ML) approach using only Colombian isolates and one sylvatic strain to root the tree (Figure 1), clearly shows two major clades, one involved mainly viruses isolated between years 1982 to 1988 and the other one mostly viruses isolated since 1990.

**Figure 2:**
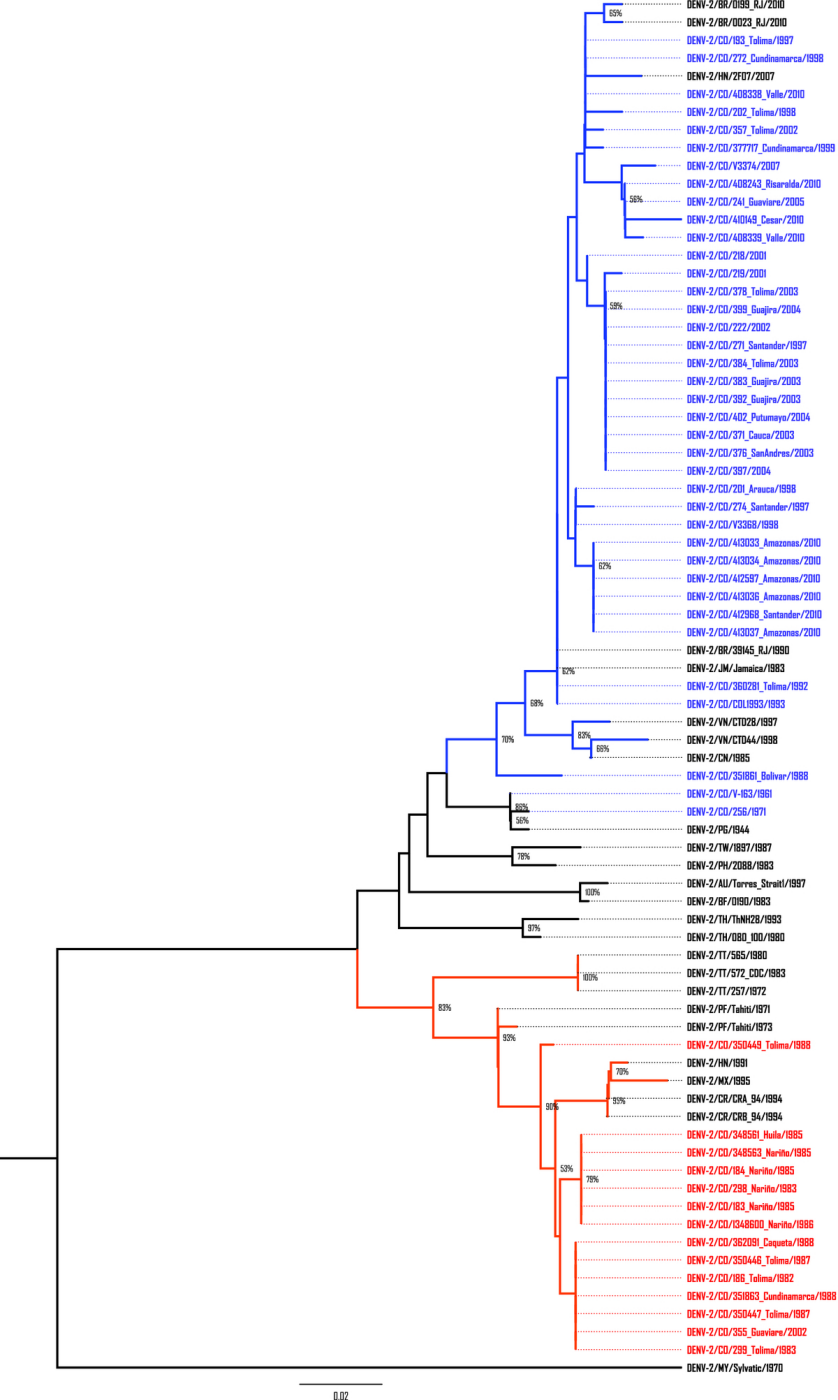
**Evolutionary relationships of DENV-2**. Phylogenetic tree of 52 DENV-2 Colombian isolates and 24 worldwide previously reported was computed using the Maximum Likelihood method. Nodes with significant Neighbor-Joining bootstrap values (≥ 5)are indicated. The tree is drawn to scale, with branch lengths in the same units as those of the evolutionary distances used to infer the phylogenetic tree. American genotype (Subtype V) and Colombian isolates belonging to this clade are show in red. American/Asian genotype (Subtype IIIb) and Colombian isolates belonging to this clade are show in blue. The tree is rooted using one sylvatic sequence.

In order to associate those clades, the ML tree was reconstructed using all previously defined DENV-2 subtypes [Subtype I (Asian II), Subtype II, Subtype IIIa (Asian I), Subtype IIIb (American/Asian), Subtype IV (Cosmopolitan), Subtype V (American) and Sylvatic genotype] (Figure 2). According to the Akaike Information Criterion (AIC), the model that better fitted the data was the Tamura-Nei equal transversion frequencies (TrNef) model. Fourteen Colombian isolates (13 reported for the first time and 1 previously described) grouped into the Subtype V clade (American genotype) close to the oldest strains from Tahiti (DENV-2/PF/Tahiti/1971; DENV-2/PF/Tahiti/1973), Trinidad (DENV-2/TT/257/1972; DENV-2/TT/565/1972; DENV-2/TT/572_CDC/1983) and the latest detected in Central (DENV-2/HN/1991; DENV-2/CR/CRA_94/1994; DENV-2/CR/CRB_94/1994) and North America (DENV-2/MX/1995). Thirteen out of these Colombian viruses were isolated between 1982 and 1988. Interestingly, although the most recent detection of American genotype was in Peru in 1996, we found one virus isolated in 2002 (DENV-2/CO/355_Guaviare/2002) belonging to this subtype. To discard cross contamination, RNA of the sample was newly obtained and re-amplified.

On the other hand, 35 Colombian sequences (34 novel and 1 previously reported) isolated between 1992 and 2010 belong to Subtype IIIb (American/Asian genotype) near to DENV-2/JM/Jamaica/1983, the putative first virus of this genotype introduced into the Americas. Subtype IIIb is divided in two clades, one representing the Asian viruses and the other one comprising the American isolates. Interestingly, one Colombian strain (DENV-2/CO/351861_Bolivar/1988) grouped into the Asian clade, separated from the remaining isolates. In order to confirm this result, RNA was extracted and amplified again. Once reconstructed, the tree showed the same topology. Nevertheless, the bootstrap support for this node is under 50%.

Two isolates from 1961 and 1971 (DENV-2/CO/V-163/1961; DENV-2/CO/256/1971) match to the 1944 New Guinea strain (Subtype I/Asian II genotype). However, it was not possible to establish the origin of those viruses.

The fragment analyzed in our study corresponded to the carboxyl terminus of envelope (E) gene, covering amino acid 422 to 495 of the E protein. As previously reported, all the American isolates have Valine (V) at the position 485, whereas Asian strains have Isoleucine at the same position [23]. On the other hand, all the sequences belonging to the Genotype IIIb have Valine at the position 484 and Alanine at the 491 (only one Colombian isolate has Valine at this position), while Genotype V isolates (all of them) have Isoleucine and Valine respectively.

**Figure 3:**
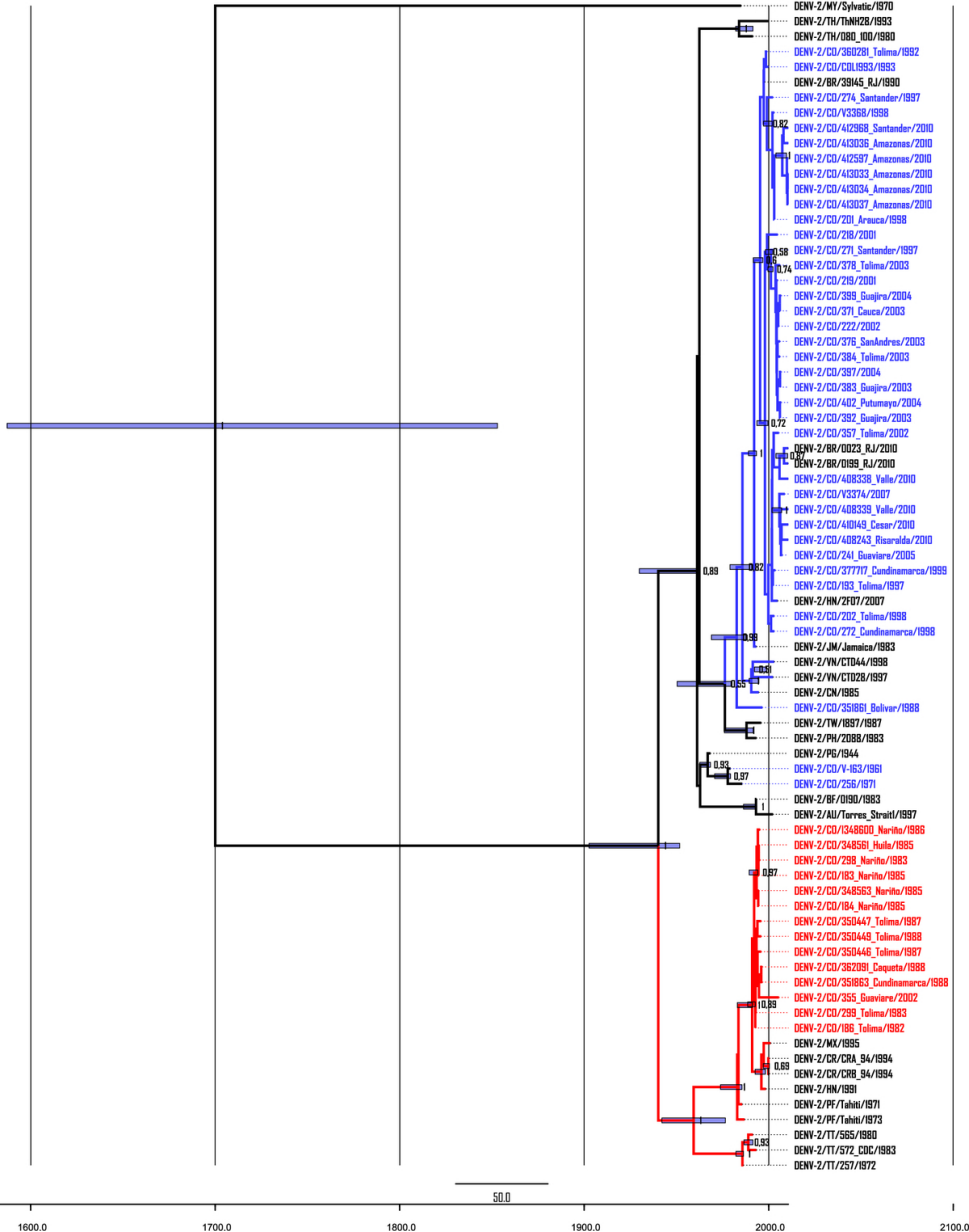
**Molecular Clock of DENV-2**. DENV-2 divergence time was estimated using year of isolation (scale used in the tree) as calibration points under the strict molecular clock model using GTR + Γ + I parameters. Posterior Probability (PP) values are indicated for each node, and the blue bars represent the extent of the 95% highest posterior density (HPD) intervals for each divergence time. American genotype (Subtype V) and Colombian isolates belonging to this clade are show in red. American/Asian genotype (Subtype IIIb) and Colombian isolates belonging to this clade are show in blue.

Using Bayesian inference and according to the 95% highest posterior density (HPD) under the strict molecular clock model, the root of the tree including sylvatic strain is placed around 270 years ago and the substitution rate was 6.6 × 10^4 ^substitutions per site per year, close to the previously reported [20,36-38]. Topology of the tree (Figure 3) shows two well supported clades representing American genotype (PP = 0.99) and the Asian/American genotype (PP = 0.99). Again, all but one (DENV-2/CO/355_Guaviare/2002) of the Colombian strains isolated between 1982 and 1988 fell into the subtype V, while those further isolated until 2010 get into the subtype IIIb. The phylogeny demonstrates a huge sustained spread of viruses all over the country, especially during the last 10 years. In fact since the year 2000, Colombian strains have been evolving in different clades, mostly clustered by the time of isolation. Interestingly, during the last epidemic in 2010, at least two different lineages had been circulating one in different localities and one almost exclusively at the Amazonas department.

## Discussion

Between 1950 and 1960, the Pan American Health Organization (PAHO) *Aedes aegypti *eradication program to fight urban yellow fever was successful to suppress dengue transmission [5]. By the year 1952 *Aedes aegypti *was virtually eradicated from Colombia, and only few cases of Dengue were reported on the Magdalena valley [5,30,31]. Unfortunately, predictions made by Dr. Hernando Groot about the real impact of dengue in the Americas were ignored and the implementation of these eradication campaigns were abandoned by the late 60's and the subsequent decades, leading the mosquitoes to proliferate and spread all over the American continent [30]. Dengue syndrome re-emerged and rapidly became the most important infectious viral disease in the Americas [1,4,5,30,31]. Since then, all DENV serotypes have been detected, being DENV-2 perhaps the most important in terms of morbidity and mortality [1,4,5,30,31].

We have reconstructed the phylogenetic history of DENV-2 in Colombia and reported for the first time the distribution of genotypes across time. Large epidemics of DENV-2 were first occurred in the Caribbean Islands, starting in Trinidad & Tobago (1953), following by Curação and Haití (1968) [1,5]. First outbreaks of DENV-2 reported in mainland, probably as a spillover from the islands, occurred in French Guiana (1970) and Colombia (1971) [1,5,30,31]. For about 10 years, the virus was reported only in Colombia where it was generating DF until 1981, when this serotype was first reported in Cuba and Jamaica [1,5,8]. Our study clearly demonstrates that Colombian DENV-2 isolated up to 1988 belongs to a well supported clade, grouped with strains previously defined as Subtype V (American genotype) [2,12,13]. One of the most significant issues of dengue history in the Americas is perhaps the first DHF outbreak occurred simultaneously in Cuba and Jamaica in 1981 [1,8,23,39,40]. Further studies demonstrated that DENV-2 involved in this severe epidemic belonged to a different genotype very close to previously characterized Asian strains [2,12,23,39,40]. This new Asian-American virus (currently known as Subtype IIIb) generates a well supported clade, nested Jamaica strains above (DENV-2/JM/Jamaica/1983) and Vietnam and China as the origin of subclade (DENV-2/CN/1985; DENV-2/VN/CTD44/1988; DENV-2/VN/CTD28/1997). Thirty-five (35) out of the 36 Colombian viruses isolated after year 1990 fell into this clade, demonstrating the spread of the Asian- American genotype all over the country during the last 20 years. Interestingly, the introduction of this subtype clearly coincides with the first official report of DHF at the end of 1989 (Puerto Berrio, Antioquia) and the sustained increase of severe cases observed during the next years [30]. Two major explanations have been suggested for DHF to occur. The antibody dependent enhancement (ADE) theory proposes the rise of severity as a result of a secondary heterologous infection, essentially in hyperendemic areas [7]. However, in Colombia the 4 serotypes were circulating already (DENV-3 in 1975, DENV-1 since 1978, DENV-4 since 1983) and yet, there were not DHF cases reported even in those localities were co-circulation of at least 2 serotypes was noted. On the other hand, the sudden increase in DHF cases after the introduction of the Subtype IIIb in the Americas (probably in Jamaica in 1981) supports the idea of the emerging of virulent strains (hemorrhagic strains) and replace of the less aggressive native American genotypes [2,9,10,12]. The marked split showed in our study between the isolates obtained before and after the appearance of DHF, clearly agrees with the second hypothesis, although the first one can explain the high incidence of severe dengue currently observed in some hyperendemic localities with co-circulation of serotypes others than DENV-2. In fact, during the last epidemic in Colombia (2010) DENV-1 and DENV-2 were isolated in high proportion equally in both DF and DHF cases but secondary infection was not demonstrated. Moreover, all four serotypes were detected in fatal cases, even though DENV-2 was the most frequent [32]. All together, these findings suggest that hyperendemicity summed to increased virulence are both decisive for DHF maintenance, more than two separate factors [2,9,10,12].

The introduction of the DENV-2 American genotype in Colombia is easy to explain, considering that the *A. aegypti *eradication programs failed in the Caribbean coast, leading the mosquito spread from Maracaibo (Venezuela) to Maicao (La Guajira, Colombia) in 1968 [30,31]. By the year 1971, the entire Colombian Atlantic coast was re-infested, including most of the important ports located in Barranquilla (Atlántico) and Cartagena (Bolivar), which maintained major commercial trades with the Caribbean islands where the virus was already established.

More difficult to explain is the replacement event of the American genotype by the Asian- American [2,12]. Since the introduction of Subtype IIIb, American had been detected only in few cases during the middle 90's in Central America and as late as 1996 in Peru [41]. The replacement and extinction of genotypes have been described as a stochastic event occurring during periods of depletion in mosquitoes population or low number of susceptible hosts [20,38,42-46]. In the present study we found one virus isolated in 2002 (DENV-2/CO/355_Guaviare/2002) placed inside the Subtype V (American genotype), indicating perhaps that the genotype is not extinct. Although differences in fitness have not been surely demonstrated (see below), it is possible for the Asian genotypes to hold a higher transmission pattern, restricting the "native" virus to low circulation dynamics and probably causing only subclinical (undetectable) infections. On this matter, it is important to notice that the samples collected come from the surveillance system and belong to symptomatic patients. Therefore, the opportunity to isolate this genotype again is even lower.

There are two major pressures affecting DENV evolution process. One is the attachment to a susceptible cell, leading to entry by membrane fusion and the other is the host immune response [18,20,38,47-51]. The envelope (E) protein is involved in both processes and therefore the most representative to infer adaptation patterns. In fact, Weaver et. al. had demonstrated the constrained effect occurring in virus obligated to alternate between invertebrate vector and vertebrate host [52,53]. Nevertheless, this effect is possible reduced when transmission rates are very high (hyperendemic areas) in human hosts [29]. On the other hand, positive selection on some DENV-2 genotypes had been previously inferred into immunogenic zones of E protein, specifically in amino acids 91, 129, 131 and 491, indicating perhaps a way for immune response evasion [18,20,23,49]. According to our results and as previously reported, all the American isolates have Valine at the position 485, whereas Asian strains have Isoleucine at the same position [23]. Interestingly, Valine at 484 and Alanine at 491 were conserved all over the Genotype IIIb, while Genotype V isolates have Isoleucine and Valine at the same positions clearly resembling the ancestral state observed in sylvatic strains (Malaysia, DENV-2/MY/Sylvatic/1970) [54]. Although the impact of this phenotypic change (if any) remains to be determined, but it strongly suggests a positive selection process acting over the E protein [49,50].

Evolution dynamics of DENV-2 is affected by several factors. Because of the lack of proof-reading activity of RNA-dependent RNA-polymerase, RNA viruses usually present higher mutation rates than DNA viruses [47,55-57]. Nevertheless, arboviruses (as mentioned above) are subject to a trade-off effect when they alternatively replicate in humans and mosquitoes [52,53]. In fact, Holmes had demonstrated that arboviruses (in general) generate more deleterious mutation than other RNA viruses. [51]. As a consequence, susceptible human populations together with vector densities might lead different evolution patterns in distinct geographic areas. Colombia is perhaps one of the most highly endemic countries in the Americas region, with a current co-circulation of the four DENV serotypes and 75% of the territory having elevated rates of *A. aegypti *infestation [31,32]. Moreover, by the year 2010, 157,152 cases of dengue were confirmed including 9.482 corresponding to DHF with 2.28% of lethality [32]. During this time, 662 viruses were isolated and 40.4% were identified as DENV-2. In spite of the constrained effect, our results of the Bayesian analysis clearly show an intense evolution process, supported by the different clades generated since the first circulation. According to the tree, Subtype IIIb Colombian isolates fall into at least 3 clades or "lineages" especially well defined after the year 2000. One clade put together most of the samples collected between 2001 and 2004. The second clade groups mainly viruses isolated in 2005 (DENV-2/CO/241_Guaviare/2005), 2007 (DENV-2/CO/V3374/2007) and 2010 (DENV-2/CO/410149_Cesar/2010; DENV-2/CO/408243_Risaralda/2010; DENV-2/CO/408339_Valle/2010; DENV-2/CO/408338_Valle/2010). Interestingly, there is a third clade clustering almost exclusively with strains from Amazonas department (only one from Santander) isolated during the last epidemic (2010). Until 2009, there was not dengue transmission in the Amazonas because of the small *A. aegipty *population, and only sporadic cases of imported infection had been notified [30]. Nevertheless, in 2010 the number of dengue patients significantly increased in Leticia, the capital city of Amazonas [32]. Epidemiological surveillance system let us confirm that most of that reported cases came from the neighbor Peruvian city of Iquitos, where a dengue outbreak was already taking place. Together, these results demonstrate the establishment and co-circulation of different lineages of the Asian/American genotype during the last decade, and the entrance of a new one during the last Colombian epidemic.

In conclusion, our phylogenetic reconstruction suggests the circulation of DENV-2 American Subtype V in Colombia for about 20 years, until the early 90's when the Asian/American Subtype IIIb replaced it. Although the first entry and subsequent establishment of this new genotype clearly coincide with the emerging and increase of severe DHF, there is no formal evidence of enhanced virulence on this genotype. On the other hand, during the last 20 years Subtype IIIb has been evolving locally and co-circulation of different clades is observed. In fact, introduction of a new "lineage" probably from Peru to the Colombian Amazon region is strongly supported. Even with the lack of viral pathogenic markers certainly documented, it is compelling that the clinical manifestation of dengue infection has changed. Atypical signs such as viscerotropism or encephalitis are becoming more recurrent and lethality rates are increasing in hyperendemic countries including Colombia. Therefore, control programs should include the surveillance of potentially pathogenic DENV genotypes together with mosquito control and people education campaigns.

## Methods

### Virus strains

DENV-2 strains used in this study were obtained from the virus collection of the National Health Institute (INS, Virology Lab, Bogotá, Colombia), and comprised 48 isolates from different outbreaks, epidemics and routine epidemiological surveillance. Clinical samples were collected between 1982 and 2010 from different localities all around the country, so they represent most viruses circulating in Colombia during the last 30 years (Table 1). All viral stocks were inoculated on C6/36 *Aedes albopictus *cells growing in Eagle's minimal essential medium (E-MEM) supplemented with 2% fetal calf serum (FSC). After 10 days of incubation at 28°C, monolayer was disrupted and supernatant was then recovered by centrifugation and stored at -80°C until use. The remained cells were washed with Phosphate Buffer Saline (PBS) and dripped on slides; after fixed in cool acetone, slides were incubated with monoclonal antibodies (anti-DENV-1 to anti-DENV-4, kindly donated by CDC, Puerto Rico) for one hour, washed with PBS and incubated again with a fluorescent conjugated antibody. Additionally, DENV-2 serotype confirmation was done by reverse transcription polymerase chain reaction (RT-PCR) using specific primers [33].

### Viral RNA extraction, RT-PCR and sequencing

Cell culture supernatants were used to extract viral RNA using QIAamp Viral RNA Minikit (Qiagen, Germany) following manufacturer's instructions. Briefly, 140 μl of each supernatant was placed into 560 μl of AVL buffer with 5.6 μl of carrier RNA and mixed with ethanol (96-100%) before passed through a column by centrifugation. After washing with buffers AW1 and AW2 RNA was finally eluted with 60 μl of AVE buffer and stored at -80°C until use. Five micro liters from each RNA extraction were used as template in a one step RT-PCR reaction (Qiagen, One-Step RT-PCR kit) as previously described [33]. Primers used [DEN2S1871 (5'-TAGCAGAAACACARCATGGNAC-3') and DEN2AS2622 (5'-CAATTCTGGTGTTATTTGYTTCCAC-3')] were designated to amplify 751 bp from the joining region E/NS1. Reactions were evaluated in 1% agarose gels stained with ethidum bromide and negative reactions were subjected to nested PCR as previously reported [29,33], using specific nested primers DEN2S2042 (5'-CAGTCAACATAGAAGCAGAACC-3') and DEN2AS2549 (5'-GCYGAAGCTAGTTTTGAAGGGG-3')]. Nested PCR was evaluated in 1% agarose gel stained with ethidium bromide.

Amplified products (from RT-PCR or nested PCR) were purified using QIAquick PCR Purification Kit (QIAGEN, Germany) and then used as template for sequencing reactions using the ABI Prism Dye Terminator Cycle Sequencing Ready Reaction Kit (Applied Biosystems, Foster City, CA) [29,33]. A total of 224 bp [corresponding to carboxyl terminus of envelope (E) gene] from 48 new sequences were compared with 28 previously sequenced strains from all over the world, available in GenBank. Consensus sequences were aligned using the program CLUSTAL W included in MEGA package version 4.0 [34,35].

### Phylogenetic analyses

Phylogenetic trees were reconstructed with the Maximum Likelihood (ML) methods incorporated in the Paralleled and Integrated Framework for Phylogenetic Inference with Automatic Likelihood Model Selector (PALM) program, which combines Clustal W, PhyML, MODELTEST, ProtTest and others in one interface. [34,35,58-60]. Statistical significance of tree topology was assessed with a bootstraping with 1000 replicates. Obtained trees were visualized using the FigTree 1.2.2. program. All the ML and MODELTEST parameters obtained are available upon request.

### Substitution rates and molecular clock

In addition, estimated rate of evolutionary change (nucleotide substitutions per site per year) and tree root age was obtained with the program BEAST (Bayesian Evolutionary Analysis by Sampling Trees) [61], which uses Bayesian Markov Chain Montecarlo (MCMC) algorithms combined with the chosen model and prior knowledge of sequence data to infer the posterior probability distribution of phylogenies [61-65]. We analyze the data using the year of isolation as calibration points to estimate divergence time in years. Rate variation among branches was inferred under the strict molecular clock model, whereas substitution rate among sites was calculated with the General Time-Reversible model (GTR) combined with the gamma parameter and proportion of invariant sites (GTR + Γ + I) model. MCMC was run for 10,000,000 steps and sampled every 500 steps and the 10,000 first steps of each run were discarded. BEAST format files were obtained in the provided BEAUti graphical interface and the trees were visualized with the FigTree 1.2.2. program. Finally, statistical analyze were carried out in the Tracer package [61].

## Competing interests

The authors declare that they have no competing interests.

## Authors' contributions

JAM contributed to the experimental design, carried out the experiments and phylogenetic and molecular clock analysis, and wrote the manuscript. JAUC contributed to the experimental design, carried out the experiments and provided a critical review of the manuscript. CD participated in the experimental design, contributed to the interpretation of data and the critical review of the manuscript. GJR contributed to the experimental design and provided a critical review of the manuscript. JAS contributed with phylogenetic and molecular clock analysis and BEAST running and provided a critical review of the manuscript. AT conceived the experimental design and provided a critical review of the manuscript. JCGG conceived the study, participated in its design and coordination and provide a final review of the manuscript. All authors read and approved the final version of the manuscript.
